# The Effect of Educational Intervention Based on Theory of Planned Behavior Approach on Complementary Feeding: A Randomized Controlled Trial

**DOI:** 10.1155/2023/1086919

**Published:** 2023-01-18

**Authors:** Qonita Rachmah, Junaida Astina, Dominikus Raditya Atmaka, Leli Khairani

**Affiliations:** ^1^Department of Nutrition, Faculty of Public Health, Universitas Airlangga, Jl. Mulyorejo Kampus C, Surabaya 60115, Indonesia; ^2^Center for Health Education, Counseling and Empowerment, Universitas Airlangga, Indonesia; ^3^Food and Nutrition Program, Faculty of Allied Health Sciences, Chulalongkorn University, Pathum Wan, Bangkok 10330, Thailand; ^4^Faculty of Nursing, Chulalongkorn University, Pathum Wan, Bangkok 10330, Thailand

## Abstract

Complementary feeding should be given to infants at 6 months in addition to breastmilk. Mothers' knowledge and behavior in giving adequate complementary feeding are crucial to prevent malnutrition risk. During the pandemic, conventional nutrition education cannot be maintained and could lead to decreased mothers' knowledge. This study is aimed at analyzing the effectiveness of nutrition education using online digital platforms (WhatsApp) to improve a mother's behavior in providing nutritious complementary food based on the theory of planned behavior approach. This was a quasiexperiment with one pretest and posttest design group in the form of education and counselling. Ten educational sessions were developed to improve one or more TPB constructs. Media used for education are PowerPoint, text description, posters, and video tutorials; it is implemented by sending materials through the WhatsApp application. Using 80% power, the sample size was calculated for 155 subjects. Subjects were recruited through the accidental sampling method. Data was collected by the online method using a validated open-ended self-developed questionnaire for knowledge, while attitude, subjective norms, intention, and self-efficacy were measured using a Likert-scale questionnaire, where participants rated the strength of their belief that they could engage in a specific task. The paired *t*-test was used to analyze the difference in outcomes measured. The response rate of this study was accounted for at 77.5%. The mean age of mothers was 28.2 years old; most of them were university graduates (80.2%) and working as private sector workers (40.0%). The average child's age was 6.6 months old. 78.2% of children were exclusively breastfed. Our study revealed that 10 sessions of nutrition education and counselling covered over 8 days increased the mother's knowledge (60.0 ± 15.5 vs. 80.3 ± 15.0, respectively, before and after education; *p* < 0.005) and resulted in psychological changes including mother's attitude (64.3 ± 4.9 vs. 65.8 ± 3.9), subjective norm (3.76 ± 0.9 vs. 3.87 ± 1.0), perceived behavioral control (3.78 ± 0.9 vs. 4.12 ± 0.12), self-efficacy (63.3 ± 22.5 vs. 77.5 ± 19.2), and intention toward giving nutritious complementary feeding (4.11 ± 1.0 vs. 4.30 ± 0.9; *p* < 0.005). WhatsApp nutrition education proved to be effective in improving the mother's knowledge and behavior in providing nutritious complementary food; thus, it has potential for use. In the future, the Ministry of Health from the district to the national level could implement this type of education as an alternative of conventional nutrition education through scheduled classes.

## 1. Introduction

The first 1000 days of life is the golden period of child development where nutritional adequacy is needed for optimal child growth and development [1]. Currently, 3 out of 10 Indonesian children are stunted, and the national government also prioritizes the stunting alleviation program to decrease stunting up to 14% in 2024 [2]. Stunting also brings a long-term negative impact such as decreased intelligence (IQ); increased risk of noncommunicable diseases, i.e., cardiovascular diseases and diabetes; decreased productivity; and loss of job opportunities that have an impact on economic status [3]. Apart from stunting, 45% of under-five mortality in the world is reported as a direct or indirect impact of malnutrition and severe malnutrition [4]. This result is reinforced by the fact that 67% of the under-five mortality rate was due to improper diet such as not getting exclusive breastfeeding, early or late complementary feeding, incomplete and unbalanced nutritional composition, and also unhygienic foods [5].

During 0-6 months, exclusive breastfeeding is proven to optimize the baby's intake because, at that time, the baby's digestive system is still undergoing refinement until it is ready to receive food at the age of >6 months [6]. After 6 months to 24 months, breastfeeding only is not sufficient; thus, complementary feeding should start to be given to babies when they approach 6 months old [7, 8]. It is important that complementary foods are given to complement the nutritional needs of babies which are increasing along with their growth and development.

In practice, parents of toddlers who experience malnutrition based on the Weight-for-Age*z*-score (WAZ) and Height-for-Age*z*-score (HAZ) indicators will be given nutrition education and counselling at the *Posyandu/*integrated health post at the village level [2], but the COVID-19 pandemic which lasted more than a year caused the *Posyandu* to stop operation; hence, nutrition education could not be optimized. In addition, currently, the digitalization era is growing fast, and digital-based nutrition education innovations must also be developed. Previous literature on multifaceted digital-based nutrition guidance system via the internet, email, and text message addresses modifiable factors linked to the early onset of childhood obesity and supports healthy growth [9]. This emphasizes the need to develop digital-based nutrition education.

Research in Jakarta, Indonesia, showed 17.1% of mothers sought information about complementary foods through electronic media, the remaining 30% from health workers, 22% from *Posyandu* health cadres/volunteers, and 18.6% from families [10]. Interestingly, a qualitative study in America in 2020 found that online resources are currently used by mothers, especially those in low-income groups, as the main source of seeking nutritional information, not health workers [11]. Therefore, online-based nutrition education is a channel that needs to be considered in its use to provide nutrition education with relevant scientific-based evidence. Due to a mother's higher level of nutritional knowledge, she avoids giving less nutritious food to her child, putting more trust in knowledge of health nutrition. The mother's level of knowledge on nutrition affects children's eating habits [12].

**Figure 1:**
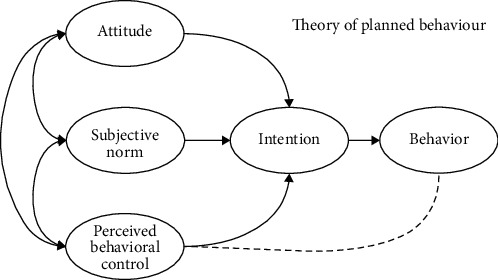
Construct of theory of planned behavior [15].

It is widely known that nutrition education is a part of nutrition intervention that has a long-lasting impact in changing behavior as well as cost effectiveness [13]. The theory of planned behavior (TPB) is one type of behavioral change theory that is considerably used in nutrition education [14]. This theory successfully predicted and demonstrated several health behaviors ranging from breastfeeding and substance use to smoking, etc., and it explains that behavior depends on the person's motivation and ability to do specific health behaviors.  Figure 1 portrays the TPB construct that addresses a behavior construct from several aspects including “attitudes,” “subjective norm,” “perceived behavioral control,” and “intention.”

A previous randomized controlled experimental study showed that a *WhatsApp midwife breastfeeding support line* among 100 postpartum mothers significantly increased mothers' breastfeeding self-efficacy scale [16]. In addition, Indonesia counted as the fourth largest WhatsApp audience with a total of 68.8 million users; therefore, it has a high potential to be used as an education platform [17]. Based on the description above, this study is aimed at analyzing the effectiveness of nutrition education using online digital platforms (WhatsApp) in increasing maternal nutritional knowledge, attitudes, subjective norms, perceived behavioral control, self-efficacy, and intention to practice healthy complementary feeding.

## 2. Materials and Methods

This was an intervention study with a quasiexperiment with one pretest and posttest design group in the form of education and counselling for 8 days to improve maternal nutrition knowledge, attitudes, subjective norms, perceived behavioral control, self-efficacy, and intention to practice healthy complementary feeding.

### 2.1. Sample Size and Selection

The sample of this research was mothers who have children aged 0-24 months, selected by the convenience sampling method, a nonprobability sampling strategy in which subjects are chosen based on their ease of access. Specifically, to this study, subjects were chosen based on their willingness to join a WhatsApp session for 8 consecutive days and eligibility based on inclusion criteria. Inclusion criteria include mothers with infants/children aged 0-24 months with installed *WhatsApp* application, while exclusion criteria are mothers who were not able to join the 8-day classes. The information of nutrition education was promoted through social media, and participants who were willing to participate were informed about the intervention informed consent was obtained. The sample size was determined using a sample size calculation to compare the mean of a continuous measurement in two samples, using a *z*-statistic to approximate the *t*-statistic with the effect size calculated from the research results of Kajjura et al. [18] regarding increasing knowledge and complementary feeding through nutrition education (*n* = 104; SD = 1.06; ES = 0.24) with 95% CI and 80% power, and a minimum sample of 155 subjects was found. The study was done in Indonesia on June–July 2021.

### 2.2. Intervention

The intervention in this study consisted of a combination of 10 nutritional education sessions over 8 consecutive days based on the *theory of planned behavior* construct. Ten sessions were purposely developed based on information needed during complementary feeding which was retrieved from the 10 WHO complementary feeding principles [19], and duration of intervention was also purposively selected based on several reasons; i.e., (1) it gave opportunity for mothers to read all the materials given as well as follow the hands-on activity; (2) it did not take too long to prevent fatigue or loss of enthusiasm. Each session was aimed at increasing one or more constructs in the *theory of planned behavior*. The 10 nutrition education sessions are summarized in Table 1.

In detail, digital-nutrition education was delivered by threes trained educators with a minimum educational background of a master's degree in nutrition and health. Each day, subjects were given 1–2 different materials (Table 1). In its implementation, classes were started at 8 am every day during the 8-day session. One topic was delivered by one nutrition educator. Hands-on-activity sessions in this intervention were aimed at increasing the participant's involvement and also their interest in nutrition education. We used several combination media during the intervention including PowerPoints (PPT), text description, posters, and video tutorials.

### 2.3. Independent Variables

The independent variables in the study were the nutrition education intervention (utilizing WhatsApp). The nutrition education intervention would be expected to influence the key outcome (dependent variable) based on the TPB construct, i.e., knowledge, attitude, self-efficacy, subjective norms, perceived behavioral control, and intention of giving nutritious complementary feeding. It is hoped that changes in these behavioral determinants will succeed in improving infant- and child-feeding behavior. Nutrition education would hypothetically result in improvement in knowledge, attitude, self-efficacy, subjective norms, perceived behavioral control, and intention. Those dependent variables were measured using an online questionnaire. After data collection, we also do the data recheck and input the data to the statistical software.

### 2.4. Characteristics and Nutrition Knowledge

A general questionnaire was developed to obtain the child's characteristic including age, birth weight, birth length, current weight, length/height, and exclusive breastfeeding status. The parent's social-economic status (SES) data that include the parent's age, educational background, occupation, household income, and number of parity were also collected. Mother's nutrition knowledge was measured using a questionnaire that was previously validated and tested before the data collection. The questionnaire consists of 10 questions covering the topic of principle of complementary feeding, nutrient content, household serving size to measure how well the mother knows about the portion size, and literacy in reading nutrition labels. That questionnaire was developed based on the topic given during the digital nutrition education session. Every correct answer will be given a score of 10, and 0 for the incorrect. For descriptive analysis, the level of knowledge was categorized based on the total score: lack of knowledge (if total score < 60), average (if total score 60-80), and good (if total score > 80) [20].

### 2.5. Theory of Planned Behavior Construct

Outcome-related psychological data were obtained in this study, including mother's attitude, subjective norm, perceived behavioral control, self-efficacy, and intention toward giving nutritious complementary feeding. All of the psychological data questionnaires were developed as Likert-scale answers based on Bandura's guide for constructing attitude, subjective norm, perceived behavioral control, self-efficacy, and intention scales [21]. Except for self-efficacy, all variables were scored using a Likert scale from 1 to 7, in which 1 represents “strongly disagree” and 7 represents “strongly agree.” For self-efficacy, the Likert scale ranged from 0 to 100; 0 represent the lowest self-efficacy and 100 is the highest. With regard to difference from knowledge level, there was no score range or categorization for TPB construct variables.

Mothers' attitude towards complementary feeding practice was measured using a four-group questionnaire (e.g., attitude toward giving nutritious complementary feeding, attitude towards giving complementary feeding based on caloric needs, attitude toward giving packaged complementary feeding, and attitude toward online nutrition education). Similar to attitude measurement, there were three other indices including perceived behavioral control (e.g., cooking complementary feeding everyday is easy, willingness to cook complementary feeding everyday, and willingness to cook a variety of complementary feeding if they know the benefit of it). Additionally, subjective norms toward giving nutritious complementary feeding were measured using 6 indicators in eight questions, including behavioral belief strength, outcome evaluation, injunctive normative belief strength, motivation to compliance, and descriptive normative belief. Intention to give nutritious complementary feeding was also measured using three questions (e.g., intention to cook complementary feeding everyday, trying to cook complementary feeding everyday, and planning to cook complementary feeding everyday).

Data on self-efficacy to give nutritious complementary feeding practice through several barriers were gathered using a structured questionnaire. Self-efficacy to give nutritious complementary feeding practice through several barriers was measured using 10 questions that presented several barriers. The validity of the questionnaire was evaluated using the Cronbach *α*, and moderate reliability was found (0.632). The 10 questions assessing self-efficacy to overcome barriers requested participants to rate their confidence in giving nutritious complementary feeding in the following circumstances: (1) feeling stressful/fatigue, (2) baby refuses to eat, (3) having a lot household chores, (4) mother thinks she cannot cook, (5) does not know what to cook, (6) does not know how much to cook, (7) working, (8) fresh foods are limited, (9) baby experiences constipation, (10) during holiday, and (11) husband is less supportive.

### 2.6. Statistical Analyses

Before conducting analysis, normality testing using the Kolmogorov-Smirnov test was performed. Paired *t*-test was used to analyze the difference in outcomes measured between before and after the intervention. This statistical analysis has been adjusted for possible confounders such as child characteristics, SES, and household characteristics. All data analyses were performed using IBM SPSS Statistics 26.

## 3. Results

Two hundred mothers participated in the beginning of the study; however, 33 participants were dropped out due to incomplete presence during intervention and another 11 did not fill out the questionnaire completely. The response rate was at 77.5%. Hence, the analyzed result of the rest of the 155 respondents was able to illustrate the effectiveness of nutrition education intervention using a WhatsApp platform.  Table 2 presents child characteristic data. It shows that the average child age was 6.6 months.

Average birth weight was 3.052 grams and birth length was 48.5 cm which are considered as normal birth weight and length based on the WHO reference standard. Mean weight was 7.4 kg, and length/height was 66.4 cm. 51.3% of respondents had boys, and 78.2% were exclusively breastfed. According to the data in Table 2, the average mother's age in this study was 28.2 and the average father's age was 30.0. Most of the mothers were university graduates (80.2%), as well as the fathers' education (77.6%). Based on parental occupation, almost half of the mothers (40%) were working as company workers, civil servants, entrepreneurs, teachers, and freelancers while most fathers were working as company workers (51.2%) and only 1 father was not working (Table 2).

Our study revealed that the 10-session WhatsApp online nutrition education significantly increased the principle of complementary feeding knowledge. The mean score was increased more than 20 points from 60.0 to 80.3 after the intervention was obtained (*p* ≤ 0.001) (Table 3).

Table 3 also presents the change in mother's attitude towards complementary feeding practice after intervention. Attitude was scored using the Likert scale from 1 to 7, in which 1 represents “strongly disagree” and 7 represents “strongly agree.” The higher the score, the more positive the attitude presented. The overall attitude score was significantly different before and after intervention (*p* ≤ 0.001). We also did analysis in each item of attitude; in general, we divided attitude measurement into four groups, i.e., attitude towards giving nutritious complementary food, attitude towards giving complementary food based on caloric needs, attitude towards giving packaged complementary food, and attitude towards online nutrition education. Each group comprises two questions that are attitude in believing benefit and enjoyment. All questions presenting attitude show a significant increase (*p* < 0.005), except for enjoyment on giving complementary food based on caloric need. Surprisingly, we found the highest increase in the attitude towards giving packaged complementary food with an increase of around 5 points after intervention.

Table 4 shows the diverse average score of mother's subjective norm associated with complementary feeding practices. Overall, mothers' subjective norm was significantly increased after the intervention (*p* < 0.005). Comparing each item in subjective norms, the highest increase was seen in parental advice and consent to give nutritious complementary feeding. This result implies that parents could be a key person in influencing complementary feeding practice among mothers. As can be seen also in Table 4, the result of nutrition education using a WhatsApp online nutrition education in this study contributes to a significant change for the mother's perceived behavioral control (*p* < 0.005). Ten sessions of online nutrition education successfully increase the mother's perception on ease of home-made cooking and variety of complementary food for their children. A significant improvement was also seen in the mother's intention towards complementary feeding practice after intervention (*p* value < 0.002).

In addition, one of the important discoveries in this study was the increase in mother's self-efficacy in overcoming barriers during the complementary feeding period (Table 5). Several barriers that can interfere with complementary feeding practices are also presented in Table 5. Mothers have the lowest self-efficacy in giving nutritious complementary food when they are on holiday (mean = 58.6) and are more confident to give nutritious complementary food even when they think they cannot cook. The highest increase of self-efficacy was seen in overcoming baby constipation. However, in general, mothers' self-efficacy in overcoming all barriers was significantly increased after intervention (*p* < 0.005). Such barriers can be the reduction of mothers' intention to give nutritious complementary feeding.

## 4. Discussion

Our study revealed that online nutrition education using the WhatsApp platform effectively increases the mother's knowledge, attitudes, subjective norms, perceived behavioral control, self-efficacy, and intention to practice healthy complementary feeding. This result was in line with a previous study which also use WhatsApp as a platform to escalate postpartum mothers' self-efficacy in giving breastmilk [16]. The Nadimin et al. [22] study also found that nutrition education related to picky eaters through WhatsApp significantly increases mothers' knowledge about type, amount, and cooked food for the picky eater child. Another application of WhatsApp nutrition training was done by Hemmatipour et al. [23] on diabetes patients which also resulted in the improvement of triglyceride levels as well as patients' quality of life. The use of WhatsApp as a nutrition education channel is based on several reasons; i.e., it is a commonly used digital platform by the mothers in Indonesia, a lot of participants can be enrolled in a nutritional educational session, and it has cost- and time-savings as well because WhatsApp is freely available for any type of smartphone. Currently, mothers also prefer online resources to look for nutritional information instead of health workers [11].

The World Health Organization (WHO) recommends complementary feeding at the age of 6 months based on some consideration that, in general, infant growth did not improve by complementary feeding before 6 months [19, 24–26]. Starting nutrition education at the earliest age of beginning complementary feeding could be beneficial to improve the mother's literacy and behavior in giving nutritious complementary feeding. One interesting finding is the amount of exclusive breastfeeding. We found higher prevalence of exclusive breastfeeding among participants than the national prevalence of exclusive breastfeeding coverage that is 67.74% [27]. Further analysis (data not shown) revealed some reasons for not giving exclusive breastfeeding including not enough breastmilk production, mother got pregnant, mother experience mastitis, and growth faltering in infant. This is also supported by the finding that exclusive breastfeeding in developing countries tends to show an increase [28]. It is unarguable that exclusive breastfeeding is essential for child growth and development especially in the first six months, such as preventing children from contracting infectious diseases (diarrhea, pneumonia, current infection of COVID-19) and, in the long term, also preventing obesity and degenerative diseases [29].

### 4.1. Mother's Characteristics and Nutritional Knowledge

The mother's education can determine the knowledge level. A study in India, Ethiopia, and Islamabad proved that mothers with higher education were likely to initiate timely complementary feeding at 6 months [30–32]. Mothers with a higher education level were also more likely to meet the minimum dietary diversity for children aged 6-23 months [33]. Moreover, a study in Ethiopia also proved that the father's education also significantly increases the timely initiation of complementary feeding (*p* = 0.002) [32]. Parent's employment and household income were a direct factor that related to the level of food security that define child dietary intake [34]. Interestingly, an analysis from Christiaensen and Alderman [35] proposes that the mother's knowledge is mutually exclusive to household income. 53.8% of respondents had given complementary feeding to their children, while the rest had not started yet. Mothers who had not started the complementary feeding period might have poorer nutrition knowledge and therefore needed nutrition education. Based on the complementary feeding practices, 51.2% had given the right meal frequency as recommended (2-3x/day) and the snacking frequency as well. In contrast, 36.9% of respondents were still giving complementary feeding > 30 minutes which is opposite the recommendation.

We found that the WhatsApp nutrition education session successfully increases nutrition knowledge among mothers. The nutrition education curriculum in our study consists of several topics: child growth monitoring, principle of complementary feeding practices, nutritional needs of infant and young children, how to cook, feeding problems, and reading nutrition label that are divided into 8 sessions. Each session took 2 hours of education and 2 hours of counselling and/or question-answer session. Counseling and/or QA session could help participant's to better understand and pay attention to the nutrition education session. This result is in line with previous studies; i.e., a study among low-income women in Minneapolis/St. Paul proved that short-term nutrition education consisting of 4 educational sessions at 75-90 minutes each significantly increases the level of nutrition knowledge specifically related to healthy food [36]. Another study in Indonesia also found that five-month nutrition education (once in two weeks) had a significant effect on the mother's nutritional knowledge. Further, it is explained that the mother's knowledge accumulation could greatly affect family nutrition because it leads to daily behavior related to health food provision [37].

Similar to our study, two studies using an online digital platform, namely, mobile MyPlate, in which respondents got biweekly text messages of the MyPlate icon and the United States Department of Agriculture's Dietary Guidelines for 7 weeks showed an effective increase in nutrition knowledge [38], as well as online education about salt intake resulting in knowledge improvement similar to in-person education [39]. Even more, online nutrition education has also shown an indication of sustainable daily behavioral change. One study supported the hypothesis that children's nutritional status was improved by only the mother's nutritional knowledge. Such knowledge was mostly facilitated by formal education which implies the policy significance with regard to acquisition of knowledge improvement outside of school or in an informal education setting. Our study finding strengthens that informal education through an online approach is proven to be beneficial in increasing the mother's knowledge.

### 4.2. Mother's Attitude, Perceived Behavioral Control, Subjective Norm, and Intention

Apart from knowledge, physiological aspects such as attitude, perceived behavioral control, subjective norm, and intention also shape individual behavior. Based on the TPB construct, behavioral outcomes are highly determined by intention to perform a given behavior which is, in turn, predicted by three belief-based constructs: attitudes, subjective norms, and perceived behavioral control [40]. We found that all behavior constructs were significantly increased after 10 sessions of nutrition education using the WhatsApp platform. A study in Australia comprising 375 participants which also used the TPB approach explained that attitude and subjective norms had a crucial role in influencing complementary feeding decision-making. Moreover, it is explained that strong positive attitudes were likely to have intention in giving CF at 6 months (timely). A 6-month quasiexperimental study conducted in 160 mother-child pairs in Ethiopia also significantly increased knowledge, attitude, and practice towards CF [41]. Mothers who more favorably evaluate and perceive pressure from important others to introduce solids at 6 months will have stronger intentions to do so [42]. One interesting finding in our study is that compared to attitude toward giving nutritious complementary feeding, mothers' attitude towards giving commercial CF product was at the lowest score. We hypothesize that this might be due to unsupported beliefs regarding benefit of commercial CF and the number of news circulating that commercial CF products are harmful to child health.

A mother's subjective beliefs are more related to perceived social pressure. Comparing between parental, peer, and physician advice and consent regarding nutritious CF, physician advice presents the highest score, but in terms of consent, peer/friend consent presents the highest score. This result implies the different roles of mother's social environment to the CF intention. Similarly, Hamilton et al. [42] proposes that the influence of group norms which could affect mothers' intentions to introduce complementary feeding at 6 months is stronger if they perceive other mothers who they know perform the intended behavior. In our study, the mother's intention to give nutritious complementary feeding based on the WHO principle might be enhanced if they perceived that other mothers know how to do it. Different from the subjective norm, perceived behavioral control focuses more on perceptions of control matching actual control in determining behavior [40]. The highest perceived behavioral control towards giving nutritious CF was seen if mothers know the benefit of it. Hence, the nutrition education program should be focused on informing the benefit of giving balanced, nutritious CF based on the infant's and young children's need.

Prior research has found that attitudes, subjective norm, and perceived behavioral control were all significant predictors of mothers' intention to breastfeed [43]. Using the same approach, the mother's good intention toward giving nutritious CF will likely impact on CF practice behavior. A cross-sectional study involving 290 mothers in Iran explained that a mother's intention to give variety of CF was strongly determined by the mother's perception that giving a variety of CF could lead to child growth [44]. It gives strong evidence that perceived behavioral control is a strong predictor for the mother's intention. Surprisingly, mothers' intention in our study had a higher score in intention to try and planning to cook healthy complementary feeding.

### 4.3. Mother's Self-Efficacy in Overcoming Barriers to Give Nutritious Complementary Feeding

Self-efficacy to overcoming barriers to give nutritious CF was measured using a Likert scale ranging from 0 to 100, rating mothers' confidence to carry out specific tasks. Term of complementary feeding self-efficacy illustrates the mother's belief in her ability towards giving adequate and nutritious complementary feeding in terms of portion, frequency, time, and variety of foods [45]. The mother's lowest self-efficacy to give nutritious complementary feeding was when they are on holiday, followed by if mothers have a lot of household chores, when mothers feel stressful/fatigue, when mothers are working, when the baby refuses to eat, when the baby has constipation, when the husband is less supportive, and when they do not know how to cook. During the nutrition education sessions, some topics covered how to overcome barriers so the mother's self-efficacy could be improved. The analysis resulted in significant increase in overall self-efficacy and each item of self-efficacy after 8 sessions of nutrition education. For example, we teach how to read nutrition labels in commercial CF products and how it can be used during holiday. As a result, self-efficacy to overcoming barriers during holiday proved to increase with the highest score increase. Other things we teach include how to prepare and cook complementary food in a short time, how to prevent and treat constipation, etc. These might be the reason why all the mothers' self-efficacy score increased to quite a high score after the intervention. The mothers who think they have better knowledge will be more confident to overcome several barriers that might come during the period of complementary feeding.

Apart from knowledge, self-efficacy also counts as a factor that contributes to behavior [46]. A study including 88 mothers related to breastfeeding practice revealed that mothers who got 3 sessions of nutrition education have better self-efficacy than the those of the control group [47]. Self-efficacy of breastfeeding increases if mothers are confident and believe that they can successfully breastfeed [48]. One review suggests that self-efficacy has frequently been a good predictor of health behavior ([49]), which is supported by Rachmah et al. [46] in that self-efficacy to engage a specific task also has a good health-related behavior, specifically related to physical activity.

### 4.4. Strength and Limitation

We note some strengths present in our current study. To our knowledge, there is no previous study specifically using the WhatsApp chatting platform as a channel for effective nutrition education; thus, the novelty of this study can be assured. Besides, nutrition education in our study was also combined with hands-on activities that can induce and increase a mother's emotional involvement in the nutrition education session. We also analyze several physiological factors together with knowledge that can induce a better behavior related to complementary feeding practices. However, we also note some limitations such as the absence of a control group which could make stronger evidence about the effectiveness of the online nutrition education approach. Thus, we suggest a future study that includes a control group. Moreover, we suggest a future study to include mothers with various educational backgrounds (low, middle, and high level) to observe different effectivities of such an intervention.

## 5. Conclusion

In conclusion, the 10-session WhatsApp online nutrition education intervention based on the theory of planned behavior significantly increases knowledge, attitude, subjective norm, perceived behavioral control, self-efficacy, and intention to give nutritious complementary feeding among mothers. The increase of those aspects was believed to be related each other as described by the TPB construct. It is suggested to the policymakers such as the Ministry of Health to adapt and continue the nutrition education effort by integrating WhatsApp online nutrition education with the existing health promotion program. This program can also add to the previously existing classes such as pregnant-mother classes or health classes in each district. We suggest further study to use other applications or social media platforms apart from WhatsApp but still widely used by the community to compare the effectiveness of each platform. Moreover, further study could also measure health practice as the outcome of intervention.

## Figures and Tables

**Table 1:** WhatsApp/online nutrition education session based on the theory of planned behavior.

Day	Topic	Duration and activity	TPB construct
1	Growth monitoring	2-hour education and 2-hour counselling	Knowledge, attitude, and self-efficacy
2	WHO principle of complementary feeding practice	2-hour education and 2-hour counselling	Knowledge, attitude, and self-efficacy
3	How to read the label of fortified complementary foods	2-hour education and 2-hour counselling	Knowledge, attitude, self-efficacy, and subjective norms
	Hands-on activities: practice reading packaged food labels	Hands-on activity	Intention and perceived behavioral control
4	Nutrient content of foods	2-hour education and 2-hour counselling	Knowledge, attitude, and self-efficacy
	How to develop complementary feeding menu according to children's nutritional needs	2-hour education and 4-hour counselling	Knowledge, attitude, and self-efficacy
5	Hands-on activities: self-developing complementary feeding menu	Hands-on activity	Intention and perceived behavioral control
6	Prepare the first complementary feeding, cooking spices, and homemade food recipes	2-hour education and 2-hour counselling	Knowledge, attitude, self-efficacy, and subjective norms
7	Overcoming constipation in children during complementary feeding period	2-hour education and 2-hour counselling	Knowledge, attitude, self-efficacy, and subjective norms
8	Overcoming eating problems in children	2-hour education and 4-hour counselling	Knowledge, attitude, self-efficacy, and subjective norms

**Table 2:** Children and household characteristics.

*Child characteristics*	Mean (SD)	Min-max
Age (months)	6.6 (3.9)	0-24
Birth length (cm)	48.5 (2.2)	38.0-58.0
Birth weight [g]	3052 (390.2)	1800-4250
Length/height (cm)	66.4 (6.2)	53-87
Weight (kg)	7.4 (1.5)	7.8-11.6
Sex (*n*, %)		
Girls	76	48.7
Boys	80	51.3
Exclusive breastfeeding (*n*, %)		
Yes	122	78.2
No	34	21.8
*Household characteristics*	Mean (SD)	Min-max
Mother's age (years)	28.2 (3.0)	23-42
Father's age (years)	30.0 (3.9)	24-48
Mother's knowledge at baseline		
Bad	95	60.9
Average	55	35.3
Good	6	3.8
Mother's knowledge at endline		
Bad	32	20.5
Average	55	35.3
Good	69	44.2
	*n*	%
Mother's education		
Senior high school	8	5.1
Diploma	23	14.7
University	125	80.2
Mother's occupation		
Housewife	60	38.5
Civil servant	24	15.4
Company worker/employee	40	25.6
Entrepreneur	14	9.0
Freelancer	13	8.3
Teacher/lecturer	5	3.2
Father's education		
Senior high school	13	8.3
Diploma	22	14.1
University	121	77.6
Father's occupation		
Civil servant	21	13.5
Company worker/employee	83	51.2
Entrepreneur	32	20.5
Freelancer	8	5.1
Teacher/lecturer	11	7.1
Not working	1	0.6
Household income (IDR)		
<2,500,000	5	3.2
2,500,000–3,000,000	5	3.2
3,000,000–3,500,000	4	2.6
3,500,000–4,000,000	4	2.6
4,000,000–4,500,000	2	1.3
4,500,000–5,000,000	10	6.4
>5,000,000	126	80.8
Number of parity		
1	134	85.9
2	17	10.9
3	3	1.9
>3	2	1.3
*Complementary feeding practices*		
Frequency of complementary feeding		
Not yet	72	46.2
1x	4	2.6
2-3x	80	51.2
Frequency of snacking		
Not yet	72	46.2
1-2x	78	50.0
3x	4	2.6
>3x	2	1,2
Duration of eating complementary feeding		
30 minutes	39	46.4
<30 minutes	26	16.7
>30 minutes	19	36.9

**Table 3:** Change in mother's knowledge and attitude towards complementary feeding practice after intervention.

Knowledge score	Before	After	Diff	*p* value^∗^
	Mean ± SD	Mean ± SD		
Knowledge	60.0 ± 15.5	80.3 ± 15.0	20.3	<0.001
*Attitude score*				
Benefit of giving nutritious complementary food	68.6 ± 4.3	68.7 ± 5.1	0.1	0.003
Enjoyment on giving nutritious complementary food	64.2 ± 10.7	66.0 ± 6.9	1.8	<0.001
Benefit of giving complementary food based on caloric need	68.7 ± 4.1	69.1 ± 3.1	0.4	0.048
Enjoyment on giving complementary food based on caloric need	66.3 ± 6.6	65.8 ± 6.7	-0.5	<0.001
Benefit of giving homemade complementary food	65.5 ± 10.4	66.5 ± 8.0	1.0	<0.001
Enjoyment on giving homemade complementary food	63.3 ± 9.2	65.3 ± 7.5	2.0	<0.001
Benefit of giving packaged complementary food	54.4 ± 13.6	60.0 ± 10.4	5.6	<0.001
Enjoyment on giving packaged complementary food	54.6 ± 13.8	58.4 ± 9.7	3.8	<0.001
Benefit of complementary feeding online education	69.0 ± 4.6	69.8 ± 1.4	0.8	0.030
Enjoyment on complementary feeding online education	68.2 ± 5.5	68.1 ± 4.8	-0.1	0.017
Overall attitude score	64.3 ± 4.9	65.8 ± 3.9	1.5	<0.001

^∗^Statistically significant at alpha < 0.05 based on the paired *t*-test.

**Table 4:** Change in mother's subjective norm, perceived behavioral control, and intention towards complementary feeding practice after intervention.

Subjective norm score	Before	After	Diff	*P* value^∗^
	Mean ± SD	Mean ± SD		
Parental advice to give nutritious complementary feeding	3.70 ± 1.0	3.84 ± 1.1	0.14	<0.001
Parental consent to give nutritious complementary feeding	3.79 ± 1.1	3.92 ± 1.1	0.13	<0.001
Physician advice to give nutritious complementary feeding	3.72 ± 1.0	3.81 ± 1.1	0.09	<0.001
Physician consent to give nutritious complementary feeding	3.88 ± 1.0	3.97 ± 1.1	0.09	<0.001
Peers'/friends' advice to give nutritious complementary feeding	3.66 ± 0.9	3.79 ± 1.0	0.13	0.001
Peers'/friends' consent to give nutritious complementary feeding	3.80 ± 1.0	3.90 ± 1.1	0.10	<0.001
Overall subjective norm score	3.76 ± 0.9	3.87 ± 1.0	0.11	<0.001
*Perceived behavioral control score*				
Perception that cooking complementary food everyday is easy	3.36 ± 1.1	3.95 ± 1.1	0.59	<0.001
I can cook complementary food everyday if I wanted to	3.90 ± 1.0	4.21 ± 0.9	0.31	0.015
I can cook variety of complementary food I know the benefit of	4.08 ± 1.1	4.32 ± 0.9	0.24	0.021
Overall perceived behavioral control score	3.78 ± 0.9	4.12 ± 0.9	0.34	0.002
*Intention score*				
I intend to cook complementary food everyday	4.06 ± 1.1	4.29 ± 0.9	0.23	0.002
I will try to cook complementary food everyday	4.13 ± 1.0	4.31 ± 0.9	0.18	0.004
I am planning to cook complementary food everyday	4.13 ± 1.0	4.32 ± 0.9	0.19	0.002
Overall intention score	4.11 ± 1.0	4.30 ± 0.9	0.19	0.002

^∗^Statistically significant at *alpha* < 0.05 based on the paired *t*-test.

**Table 5:** Change in mother's self-efficacy in giving nutritious complementary feeding practice through several barriers.

Self-efficacy score	Before	After	Diff	*P* value^∗^
	Mean ± SD	Mean ± SD		
When I feel stressful/fatigue	61.2 ± 26.5	74.4 ± 23.1	13.2	<0.001
When baby refuses to eat	63.5 ± 26.9	78.2 ± 21.0	14.7	<0.001
When I have a lot of household chores	60.9 ± 26.2	74.5 ± 23.2	13.6	<0.001
When I think I cannot cook	66.7 ± 28.4	79.5 ± 24.0	12.8	<0.001
When I do not know what to cook	64.7 ± 27.7	79.0 ± 23.2	14.3	<0.001
When I do not know how much to cook	63.3 ± 28.2	78.4 ± 21.8	15.1	<0.001
When I work	62.8 ± 27.6	78.5 ± 23.8	15.7	<0.001
When fresh foods are limited	62.0 ± 26.6	77.1 ± 21.2	15.1	<0.001
When baby experiences constipation	65.9 ± 26.3	81.9 ± 21.7	16.0	<0.001
During holiday	58.6 ± 27.9	72.3 ± 25.0	13.7	<0.001
When husband is less supportive	66.2 ± 28.7	78.7 ± 25.2	12.5	<0.001
*Overall self-efficacy score*	63.3 ± 22.5	77.5 ± 19.2	14.2	<0.001

^∗^Statistically significant at *alpha* < 0.05 based on the paired *t*-test.

## Data Availability

The data that support the findings of this study are available on request from the corresponding author (QR). The data are not publicly available because they contain information that could compromise the privacy of research participants.
